# Aspecific binding of anti-NK1.1 antibodies on myeloid cells in an experimental model for malaria-associated acute respiratory distress syndrome

**DOI:** 10.1186/s12936-024-04944-9

**Published:** 2024-04-18

**Authors:** Emilie Pollenus, Fran Prenen, Hendrik Possemiers, Sofie Knoops, Tania Mitera, Jochen Lamote, Amber De Visscher, Leen Vandermosten, Thao-Thy Pham, Patrick Matthys, Philippe E. Van den Steen

**Affiliations:** 1 Laboratory of Immunoparasitology, Department of Microbiology, Immunology and Transplantation, Rega Institute for Medical Research, KU Leuven, Leuven, Belgium; 2 Laboratory of Immunobiology, Department of Microbiology, Immunology and Transplantation, Rega Institute for Medical Research, KU Leuven, Leuven, Belgium; 3 Laboratory for Molecular Cancer Biology, Department of Oncology, VIB, KU Leuven, Leuven, Belgium; 4 Currently at Clinical Immunology Unit, Department of Clinical Sciences, Institute of Tropical Medicine Antwerp, Antwerp, Belgium

**Keywords:** Malaria, Natural killer cells, Lung pathology, Flow cytometry

## Abstract

**Background:**

Conventional natural killer (cNK) cells play an important role in the innate immune response by directly killing infected and malignant cells and by producing pro- and anti-inflammatory cytokines. Studies on their role in malaria and its complications have resulted in conflicting results.

**Methods:**

Using the commonly used anti-NK1.1 depletion antibodies (PK136) in an in-house optimized experimental model for malaria-associated acute respiratory distress syndrome (MA-ARDS), the role of cNK cells was investigated. Moreover, flow cytometry was performed to characterize different NK cell populations.

**Results:**

While cNK cells were found to be dispensable in the development of MA-ARDS, the appearance of a NK1.1^+^ cell population was observed in the lungs upon infection despite depletion with anti-NK1.1. Detailed characterization of the unknown population revealed that this population consisted of a mixture of monocytes and macrophages that bind the anti-NK1.1 antibody in an aspecific way. This aspecific binding may occur via Fcγ receptors, such as FcγR4. In contrast, in vivo depletion using anti-NK1.1 antibodies was proved to be specific for cNK cells.

**Conclusion:**

cNK cells are dispensable in the development of experimental MA-ARDS. Moreover, careful flow cytometric analysis, with a critical mindset in relation to potential aspecific binding despite the use of commercially available Fc blocking reagents, is critical to avoid misinterpretation of the results.

**Supplementary Information:**

The online version contains supplementary material available at 10.1186/s12936-024-04944-9.

## Background

Conventional natural killer (cNK) cells constitute around 10% of human peripheral blood mononuclear cells, and induce direct cytotoxicity against infected and malignant cells [1]. In addition, they produce pro-inflammatory cytokines, such as interferon-γ (IFN-γ) and tumour necrosis factor (TNF), and chemokines, such as CC chemokine ligand 5 (CCL5). In contrast, cNK cells may also have a regulatory phenotype by producing anti-inflammatory cytokines and induce apoptosis of highly activated or stressed leukocytes [2–4]. In mice, cNK cells are characterized by the expression of pan-NK cell marker CD49b (often designated as DX5) and NKp46 and of the transcription factors T-bet and Eomes [5–8]. In contrast, in the liver, a population of T-bet^+^ Eomes^−^ DX5^−^ NK cells exists [5]. This population is CD49a^+^ (while cNK cells are CD49a^−^) and are also called liver tissue-resident NK cells. Recently, NK cell subsets have been reclassified as subtypes of innate lymphoid cells (ILCs). ILCs are considered as the innate counterparts of T cells since they lack adaptive antigen receptors, and they can be divided into three groups, namely ILC1, ILC2 and ILC3 which mirror Th1, Th2 and Th17, respectively [6]. ILC1s share markers with cNK cells such as NKp46 and T-bet, but do not express DX5 and Eomes [6–8]. ILC1s also express CD49a, a marker for tissue-residency.

*Plasmodium* parasites cause malaria, resulting in more than 200 million clinical cases and 600,000 deaths each year [9]. Most deaths are the consequence of complications such as cerebral malaria, severe malarial anaemia, acute kidney injury and malaria-associated acute respiratory distress syndrome (MA-ARDS) [10]. Infection of C57BL/6 mice with *Plasmodium berghei* strain NK65 serves as a representative model to study MA-ARDS since both infected mice and patients develop excessive pulmonary inflammation and disruption of the alveolar-capillary membranes, which are the key characteristics of MA-ARDS. The lung complication is further represented by pulmonary edema, microhemorrhages and subsequently hypoxaemia [11, 12].

The role of cNK cells in malaria is still unclear due to conflicting results in the literature [13]. Early after infection, cNK cells are thought to contribute to anti-parasitic immune responses by producing IFN-γ. However, this early IFN-γ production may also cause immunopathology. cNK cells were found to be dispensable in anti-parasite immunity in *P. berghei* strain K173-infected C57BL/6 and in *P. berghei* strain ANKA-infected BALB/c mice, while a crucial role for cNK cells was described in *Plasmodium chabaudi* strain AS-infected C57BL/6 and A/J mice [14–16]. Depletion of cNK cells with anti-asialo GM1 antibodies suggested a pathogenic role for cNK cells in experimental cerebral malaria, but these results could not be confirmed using the more specific anti-NK1.1 antibody [14, 17, 18]. Also in liver pathology, no role for cNK cells was found [19]. Therefore, using in vivo* c*NK cell depletion, the role of cNK cells during MA-ARDS development was investigated in *P. berghei* NK65-infected C57BL/6 mice.

By using the mouse anti-NK1.1 depleting antibodies, this study demonstrated that cNK cells are not involved in the development of MA-ARDS. Remarkably, by using flow cytometry on the lungs of *P. berghei* NK65-infected C57BL/6 mice, the appearance of an unknown NK1.1^+^ DX5^−^ cell population, which was not depleted by in vivo injection of anti-NK1.1 depleting antibodies, was observed. Further characterization of this population revealed that the binding of mouse anti-NK1.1 antibodies during the flow cytometric staining of these cells is aspecific, and that this population is a mixture of myeloid cells, mainly monocytes and macrophages, that upregulate FcγR4 upon infection. Staining using rat anti-DX5 and rat anti-NKp46 antibodies was specific for cNK cells and may, therefore, provide useful alternatives. Importantly, in vivo depletion using mouse anti-NK1.1 antibodies was specific for cNK cells.

## Methods

### Mice and dissections

Seven to eight weeks old C57BL/6 mice were purchased from Janvier Labs (Le Genest-Saint-Isle, France) and housed in a specific pathogen-free facility. All mice were housed in individually ventilated cages and received ad libitum high energy food (Ssniff Spezialdiäte GMBH, Soest, Germany) and water, which was supplemented with 0.422 mg/ml 4-amino-benzoic acid sodium (PABA; Sigma-Aldrich, Bornem, Belgium) after infection. All experiments were performed at the KU Leuven according to the regulations of the European Union (directive 2010/63/EU) and the Belgian Royal Decree of 29 May 2013, and were approved by the Animal Ethics Committee of the KU Leuven (License LA1210186, project P049/2018 & P084/2020, Belgium). Mice were euthanized by intraperitoneal (i.p.) injection of 100 µl of dolethal (Vétoquinol, Aartselaar, Belgium; 200 mg/ml). Blood was collected via cardiac puncture using heparinized (LEO, Pharma, Lier, Belgium) syringes and broncho-alveolar lavage was performed before transcardial perfusion to obtain the broncho-alveolar lavage fluid (BALF). Samples were processed as described previously [20]. Lungs were collected for analysis using flow cytometry or fluorescence-activated cell sorting.

### Experimental set-up

Mice were infected with *P. berghei* NK65 (Edinburgh strain [21, 22]) The disease severity of the mice was evaluated daily from day 6 dpi onwards based on parasitaemia and clinical score as described previously [20]. For the early depletion, 200 µg/mouse/day of anti-NK1.1 (mouse IgG2a, clone PK136; BioXCell, Lebanon, NH, USA) antibodies or PBS were injected i.p. at -2, 1, 4 and 7 dpi. For the late depletion, 500 µg/mouse/day of mouse anti-NK1.1 antibodies or PBS were injected i.p. at 6 dpi.

### Assessment of lung pathology

Lung pathology was quantified based on the weight of the unperfused left lung and by determination of the protein concentration in the supernatant of BALF samples using Bradford assay (Bio-Rad, Hercules, CA, USA).

### Isolation of cells from the lungs

Two different protocols were used for the isolation of cells from the lungs as described previously [20].

Protocol 1: lungs were collected in HEPES buffer (10 mM HEPES, 150 mM NaCl, 5 mM KCl, 1 mM MgCl_2_, 1.8 mM CaCl_2_; pH 7.4) supplemented with 2 mg/ml collagenase D (Sigma-Aldrich) and 0.04 mg/ml DNase I (Sigma-Aldrich). Subsequently, the lungs were homogenized in the gentleMACS™ Dissociator according to the manufacturer’s instructions (MACS Miltenyi Biotec) followed by incubation for 30 min at 37 °C. After a second processing in the gentle MACS Dissociator, cells were passed through a 70 µm nylon cell strainer. Leukocytes were isolated using a Percoll gradient. Cells were washed and resuspended in PBS + 2% FCS and live cells were counted after 1/2 dilution in trypan blue in a Bürker chamber.

Protocol 2: lungs were collected in RPMI buffer (RPMI glutamax + 5% FCS + 1% Penicillin/streptomycin) with 0.1% beta-mercaptoethanol at RT. Lungs were first minced into small chunks with scissors and then incubated for 30 min at 37 °C in digestion medium containing collagenase D and DNase I. Afterwards, tissue chunks were minced using a needle and syringe and fresh digestion medium was added for a second incubation at 37 °C for 15 min. Lung tissue was again minced with the syringe and centrifuged. The cell pellet was resuspended using 10 mM EDTA and further diluted in PBS + 2% FCS. RBC lysis was performed using 0.83% ammonium chloride/10 mM Tris and the cells were passed through a 70 µm nylon cell strainer. Cells were washed and resuspended in PBS + 2% FCS and live cells were counted after 1/2 dilution in trypan blue in a Bürker chamber.

### Staining and flow cytometry of cells

1.5–3 million cells per sample were transferred to 96 well plates and washed with PBS. Cells were incubated for 15 min at room temperature (RT) in the dark with a viability dye, Zombie Aqua (1/1000; Biolegend, San Diego, CA, USA), Zombie UV (1/1000; Biolegend) or Fixable viability dye eFluor780 (1/1000; eBioscience, Aalst, Belgium), together with Mice Fc block (MACS Miltenyi Biotec, Leiden, The Netherlands). After washing twice with cold PBS + 2% FCS + 2 mM EDTA, the cells were stained with a panel of monoclonal antibodies (Additional file 1: Table S1) dissolved in Brilliant stain buffer (BD Biosciences; Erembodegem, Belgium) for 20 min at 4 °C in the dark. In case of a competition test, incubation with panel of monoclonal antibodies is preceded by 20 min incubation at 4 °C in the dark with the antibody for which competition is tested and again washing two times with cold PBS + 2% FCS + 2 mM EDTA. After incubation with the antibody mix, cells were washed twice with PBS, transferred to FACS tubes and fixated in PBS + 0.4% formaldehyde.

100,000 or 200,000 live single cells were analysed per sample with a BD Fortessa Flow cytometer (BD Biosciences), depending on the panel (Additional file 1: Table S1). Data were analysed with FlowJo v10 software (FlowJo LLC, Ashland, OR, USA) according to gating strategies as shown in the figures or as described previously [20]. In order to calculate the absolute numbers of each cell type, the frequency of this cell type among live cells was multiplied by the total number of live cells counted using the Bürker chamber.

### Fluorescence-activated cell sorting of leukocytes and cytospin preparation

Before staining the isolated lung cells for fluorescence-activated cell sorting, purity of samples was further improved before counting. More specifically, cells were resuspended in PBS with Debris Removal Solution (MACS Miltenyi Biotec). PBS was added dropwise on top to form a lower-density layer. After centrifugation at 3000*g* for 10 min at 4 °C without brake, cells were in the pellet, while debris that accumulated in the top two phases was discarded. Next, cells were washed in PBS. Another three wash steps were performed to clean up the samples. Therefore, cells were resuspended in 10 ml of PBS + 2% FCS and then centrifuged at 300 g for 5 min at 4 °C.

Cells were stained as described above for flow cytometry (Additional file 1: Table S1). Next, 150 000 cells were sorted using a BD FACSAria Fusion (BD Biosciences) according to the gating as shown in Fig. 4. Sorted populations were T cells (CD45^+^ CD3^+^ NK1.1^−^), classical NK cells (CD45^+^ CD3^−^ NK1.1^+^ DX5^+^ CD49a^−^), macrophages (CD45^+^ CD3^−^ NK1.1^−^ CD11b^+^ MHCII^+^) and the unknown population (CD45^+^ CD3^−^ NK1.1^+^ DX5^−^ CD49a^+^).

Cytospins were prepared from the sorted populations. Therefore, 50,000 cells were dissolved in 500 µl PBS + 2% FCS and mounted on cytospin slides by cytocentrifugation for 8 min at 700 rpm using the Shandon cytospin 2 (Thermo Fischer Scientific, Aalst, Belgium). After the slides were air-dried, they were stained with Hemacolor® Rapid staining of blood smear solution 1, 2 and 3 (Sigma-Aldrich). Morphology was analysed under a 40 × lens of a Leica DM2000 LED microscope (Leica microsystems).

### Statistical analysis

The data were statistically analysed with the non-parametric Mann–Whitney U test followed by the Holm-Bonferroni correction using the GraphPad PRISM software (GraphPad, San Diego, California, USA). P-values were indicated as follows: *p < 0.05, **p < 0.01, ***p < 0.001. The horizontal black line in each group indicates the median. Statistical differences compared to the uninfected control group are indicated with asterisk above the individual data sets and horizontal lines with asterisk on top indicate significant differences between groups.

## Results

### cNK cell depletion has no effect on the development of experimental MA-ARDS

**Fig. 1:**
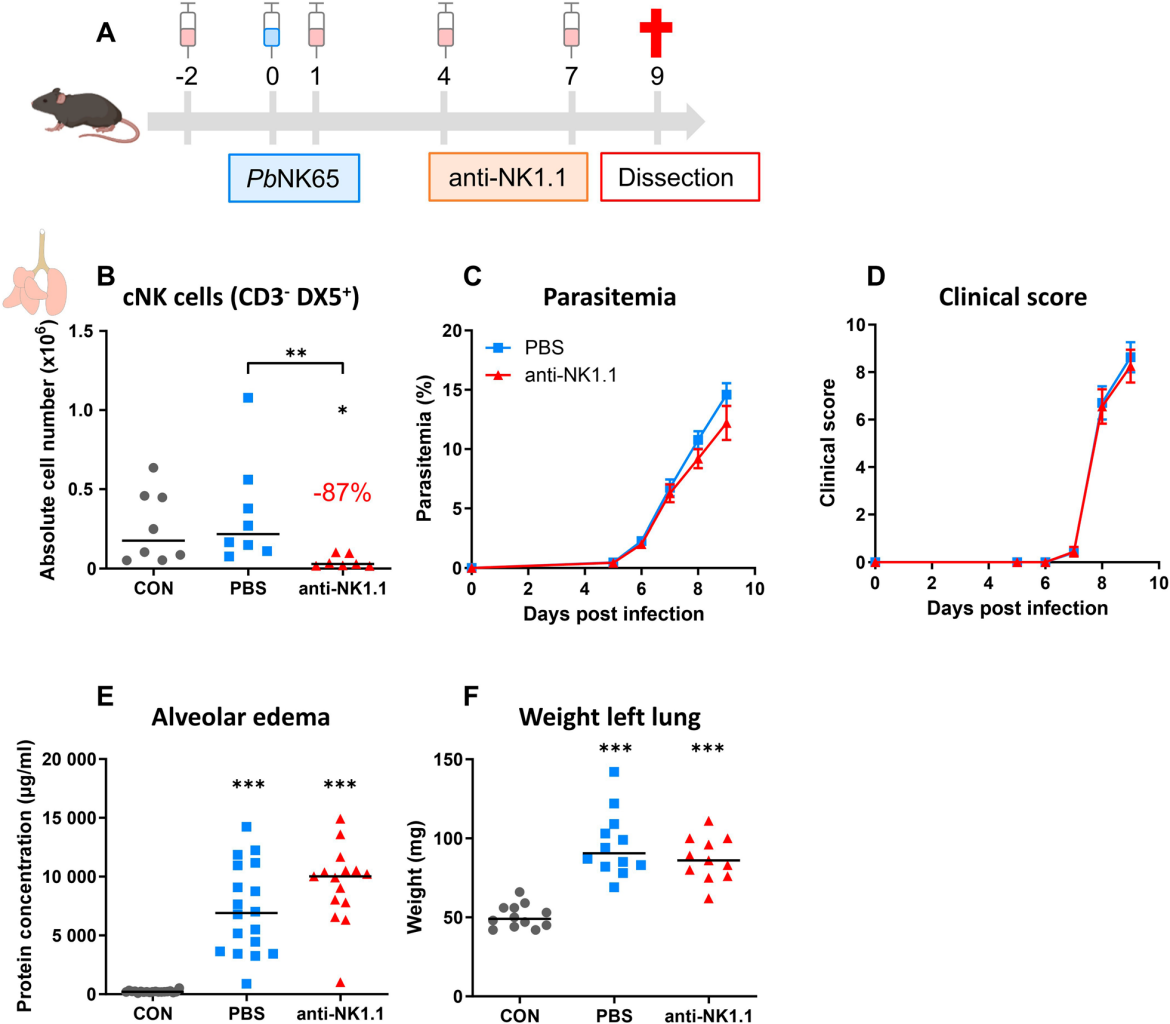
cNK cell depletion has no effect on the development of experimental MA-ARDS. C57BL/6 mice were infected with *P. berghei* NK65. Mice received 200 µg of mouse anti-NK1.1 (PK136) antibodies or PBS by i.p. injection at -2, 1, 4 and 7 dpi. Mice were dissected at 9 dpi. Leukocytes were isolated from the lungs according to protocol 1 and flow cytometry was performed. **A** Schematic representation of the experimental set-up. **B** Number of pulmonary cNK cells gated as CD45^+^ CD3^−^ DX5^+^. Data from 2 experiments. Each symbol represents data of an individual mouse. n = 8 for uninfected controls (CON), n = 8 for PBS, n = 7 for anti-NK1.1. P-values were indicated as follows: *p < 0.05, **p < 0.01, ***p < 0.001. The horizontal black line in each group indicates the median. Statistical differences compared to the CON group are indicated with asterisk above the individual data sets and horizontal lines with asterisk on top indicate significant differences between groups. **C** Parasitaemia was counted daily starting at 5 dpi using Giemsa-stained blood smears. **D** Clinical score was monitored daily starting at 5 dpi. **C**–**D** Data from four experiments. Data are represented as means ± SEM. n = 19–20 for PBS, n = 16–18 for anti-NK1.1. **E** Level of alveolar edema was determined based on protein concentration in the BALF. **F** Unperfused left lung was weighed as a second marker for pulmonary edema. Data from two (**F**) or four (**E**) experiments. Each symbol represents data of an individual mouse. n = 12–19 for CON, n = 12–18 for PBS, n = 11–15 for anti-NK1.1. P-values were indicated as follows: *p < 0.05, **p < 0.01, ***p < 0.001. The horizontal black line in each group indicates the median. Statistical differences compared to the CON group are indicated with asterisk above the individual data sets and horizontal lines with asterisk on top indicate significant differences between groups

To evaluate the role of cNK cells during MA-ARDS development, early depletion of cNK cells was performed by i.p. injection of 200 µg mouse anti-NK1.1 antibodies in *P. berghei* NK65-infected C57BL/6 mice at -2, 1, 4 and 7 dpi. The experimental design was schematically represented in Fig. 1A. The successful depletion of NK cells by anti-NK1.1 was evidenced from the 87% decrease in number of CD3^−^ DX5^+^ cells (Fig. 1B). Depletion of cNK cells had no effect on the development of MA-ARDS, since no significant differences were found in parasitaemia (Fig. 1C), clinical score (Fig. 1D) and lung pathology, based on the level of alveolar oedema (Fig. 1E) and the weight of the left lung (Fig. 1F).

### ***Appearance of an unknown NK1.1***^+^***cell population in the lungs of P. berghei NK65-infected C57BL/6 mice***

**Fig. 2:**
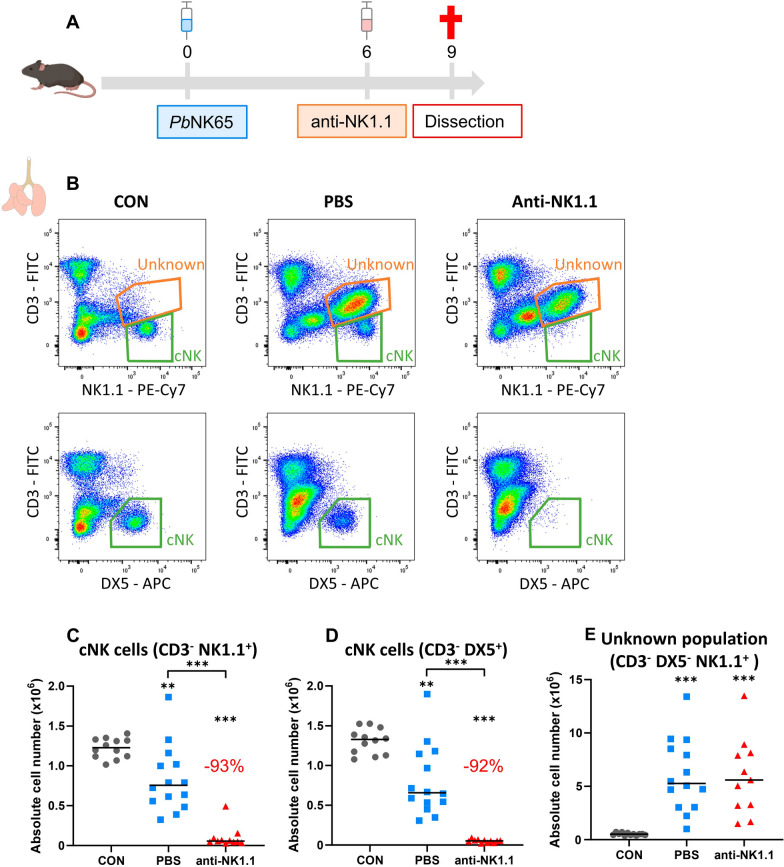
Appearance of a NK1.1^+^ population upon *P. berghei* NK65 infection that is not depleted with anti-NK1.1 antibodies. C57BL/6 mice were infected with *P. berghei* NK65. Mice received 500 µg of anti-NK1.1 (PK136) antibodies or PBS by i.p. injection at 6 dpi. Mice were dissected at 9 dpi. Leukocytes were isolated from the lungs according to protocol 2 and flow cytometry was performed. **A** Schematic representation of the experimental set-up. **B** Representative plots showing the appearance of an unknown NK1.1^+^ population (orange gate in upper row) upon *P. berghei* NK65 infection that is different from the cNK cells (green gate in upper row). Gating on DX5 instead of NK1.1 only shows the cNK cells (green gate in bottom row) (C-D) Number of NK cells present in the lungs gated as (**C**) CD45^+^ CD3^−^ NK1.1^+^ or as **D** CD45^+^ CD3^−^ DX5^+^. **E** Absolute number of unknown population in the lungs. **C**–**E** Data from three experiments. Each symbol represents data of an individual mouse. n = 12 for uninfected controls (CON), n = 14 for PBS, n = 11 for anti-NK1.1. P-values were indicated as follows: *p < 0.05, **p < 0.01, ***p < 0.001. The horizontal black line in each group indicates the median. Statistical differences compared to the CON group are indicated with asterisk above the individual data sets and horizontal lines with asterisk on top indicate significant differences between groups

Next, it was investigated whether all NK cell populations were identified using DX5 and were depleted using anti-NK1.1 antibodies in vivo. Mice were infected with *P. berghei* NK65 and late depletion of cNK cells was performed by i.p. injection of 500 µg anti-NK1.1 antibodies at 6 dpi and dissection at 9 dpi (Fig. 2A). Upon infection with *P. berghei* NK65, a CD3^−^ NK1.1^+^ population (Fig. 2B, orange gate in left and middle top panel) that is different from the cNK cells (Fig. 2B, green gate in left and middle top panel) appeared in the lungs. This population was not observed when using DX5 instead of NK1.1 (Fig. 2B, left and middle bottom panel). Late depletion by i.p. injection of anti-NK1.1 antibodies at 6 dpi (Fig. 2A), resulted in a more than 90% depletion of cNK cells, irrespectively on whether NK1.1 or DX5 was used for the identification (Fig. 2B right top and bottom panels and 2C–D). In contrast, the unknown population could not be depleted using anti-NK1.1 antibodies (Fig. 2B right top panel and 2E). Taken together, infection with *P. berghei* NK65 resulted in the appearance of an unknown NK1.1^+^ DX5^−^ cell population in the lungs, which was not depleted using anti-NK1.1 antibodies.

### ***Detailed characterization of the unknown NK1.1***^+^***cell population in experimental MA-ARDS revealed aspecific binding of the mouse anti-NK1.1 staining antibody***

**Fig. 3:**
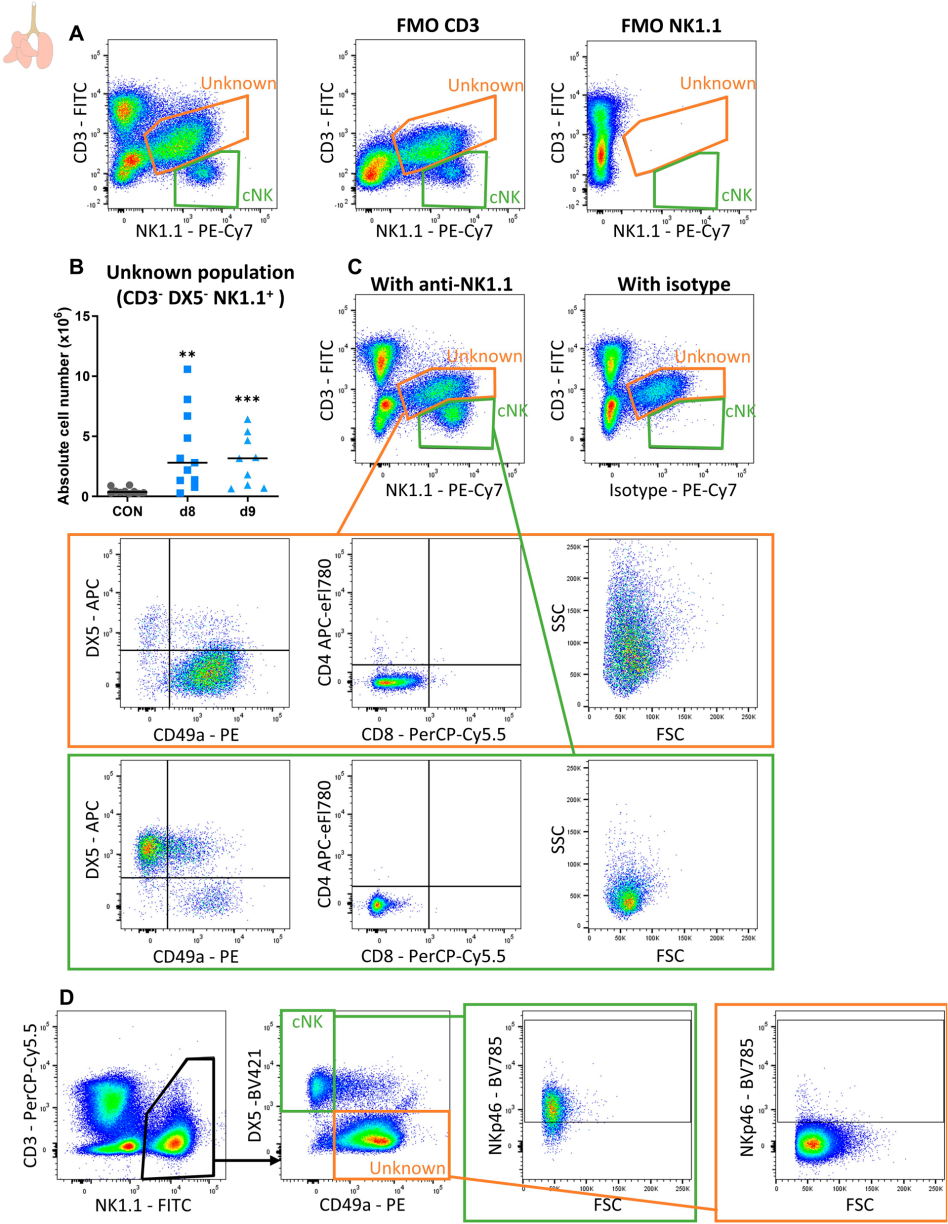
Detailed characterization of unknown population revealed aspecific binding of the anti-NK1.1 antibody. C57BL/6 mice were infected with *P. berghei* NK65 and dissected at 8 or 9 dpi. Leukocytes were isolated from the lungs according to protocol 2 and flow cytometry was performed. **A** Representative plots showing the gating of the unknown NK1.1^+^ population (orange gate) and the cNK cell population (green gate) with the appropriate fluorescence minus one (FMO) controls (without anti-CD3 (middle panel) or without anti-NK1.1 staining antibodies (right panel)) of CD45^+^ cells isolated from a *P. berghei* NK65-infected mouse at 9 dpi. **B** Absolute cell number of the unknown population in the lungs. Data from two experiments. Each symbol represents data of an individual mouse. n = 10 for uninfected controls (CON), n = 11 for d8, n = 9 for d9. P-values were indicated as follows: *p < 0.05, **p < 0.01, ***p < 0.001. The horizontal black line in each group indicates the median. Statistical differences compared to the CON group are indicated with asterisk above the individual data sets and horizontal lines with asterisk on top indicate significant differences between groups. **C** Representative plots showing the characterization of the unknown population in comparison with the cNK cells based on DX5, CD49a, CD4, CD8, FSC and SSC of a *P. berghei* NK65-infected mouse at 8 dpi. Representative plots showing staining with the isotype control antibody in comparison to staining with the anti-NK1.1 antibody. **D** Representative plots showing the expression of NKp46 on the unknown population and cNK cells isolated from a *P. berghei* NK65-infected mouse at 8 dpi

Since this unknown population appeared positive for NK1.1 based on flow cytometric analysis but could not be depleted in vivo using the same clone of mouse anti-NK1.1 antibodies, these cells were further investigated. CD3 and NK1.1 staining were evaluated using fluorescence minus one (FMO) controls, demonstrating that these cells were negative for CD3, but positive for NK1.1 (Fig. 3A). This unknown population increased in absolute number in the lungs upon *P. berghei* NK65 infection at 8 and 9 dpi (Fig. 3B). Moreover, these cells were mainly DX5^−^ CD49a^+^ (in contrast to DX5^+^ CD49a^−^ cNK cells), NKp46^−^ (in contrast to NKp46^+^ cNK cells) and CD4^−^ CD8^−^ (Fig. 3C, D). These markers could suggest that the population are ILC1s, sometimes also referred to as tissue-resident NK cells. However, forward scatter (FSC) and side scatter (SSC) showed that these cells are larger and more granular than what would be expected for most lymphocytes, such as cNK cells (Fig. 3C).

The specificity of the anti-NK1.1 antibody binding was verified by using a corresponding mouse IgG2a isotype control. Staining with the isotype control resulted in similar staining for the unknown population compared to staining with mouse anti-NK1.1 staining antibody, while cNK cells were not stained with the isotype control (Fig. 3C). These results demonstrate that the mouse anti-NK1.1 binding to the unknown population during flow cytometry staining is aspecific and that the included mice Fc block step to counter this was insufficient.

Next, an attempt to block the aspecific binding of anti-NK1.1 antibodies was performed by pre-incubation of the cells with the isotype control. Unfortunately, pre-incubation with a fluorescently-labelled isotype (mouse IgG2a) control (0.25 µg or 1.25 µg) was insufficient to block this aspecific binding of anti-NK1.1 (0.25 µg), since no shift in mean fluorescence intensity (MFI) was found for NK1.1 (Additional file 1: Fig. S1).

Overall, these data indicate that the unknown NK1.1^+^ cell population that appears in the lungs of *P. berghei* NK65-infected mice are not NK cells, and that the use of the Fc-blocking reagent does not inhibit the aspecific binding of the NK1.1 or its corresponding mouse isotype control antibody.

### ***The unknown pulmonary NK1.1***^+^***cell population in experimental MA-ARDS consists of a heterogeneous population of myeloid cells***

**Fig. 4:**
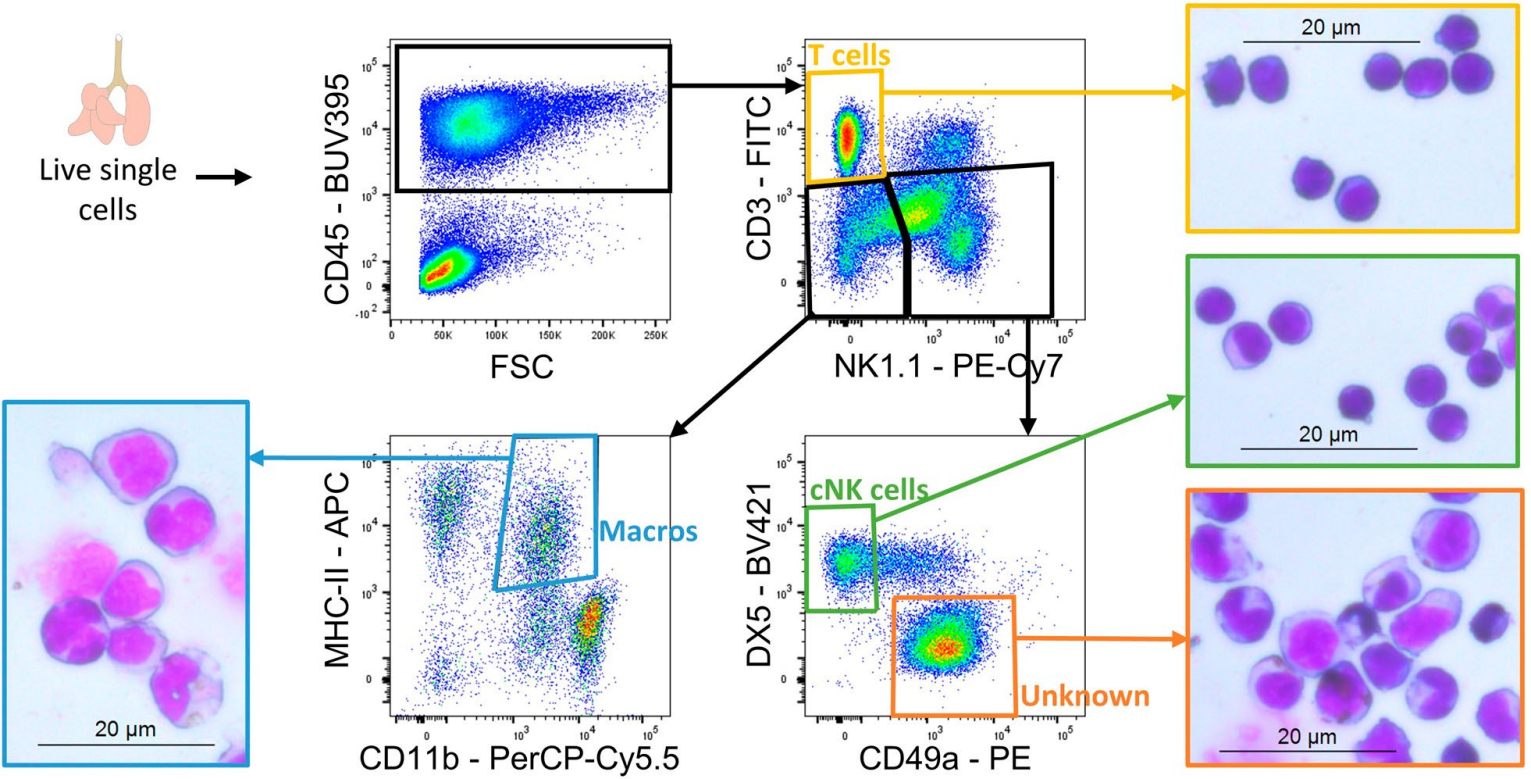
Sorting of the unknown population demonstrates a mixture of monocytes and macrophages based on morphology. C57BL/6 mice were infected with *P. berghei* NK65. Mice were dissected at 8 dpi. Leukocytes were isolated from the lungs according to protocol 2 and fluorescence-activated cell sorting was performed. Cytospins were prepared of sorted cells (T cells, cNK cells, macrophages (macros) and unknown population) to assess morphology. Representative plots demonstrating the gating strategy for the sorting and representative images of the cytospins of the sorted populations are shown

In order to identify and characterize this unknown population, these cells (CD3^−^ NK1.1^+^ DX5^−^ CD49a^+^) were sorted in parallel with T cells (CD3^+^ NK1.1^−^), cNK cells (CD3^−^ NK1.1^+^ DX5^+^ CD49a^−^) and macrophages (CD3^−^ NK1.1^−^ CD11b^+^ MHCII^+^) and cytospins were prepared (Fig. 4). Cells from the unknown population were different from the T cells and NK cells, but had a morphology similar to monocytes and macrophages. In conclusion, a heterogeneous population containing monocytes and macrophages, may be responsible for the aspecific binding of anti-NK1.1 antibodies.

### FcγR4 might be responsible for the aspecific binding of anti-NK1.1

**Fig. 5:**
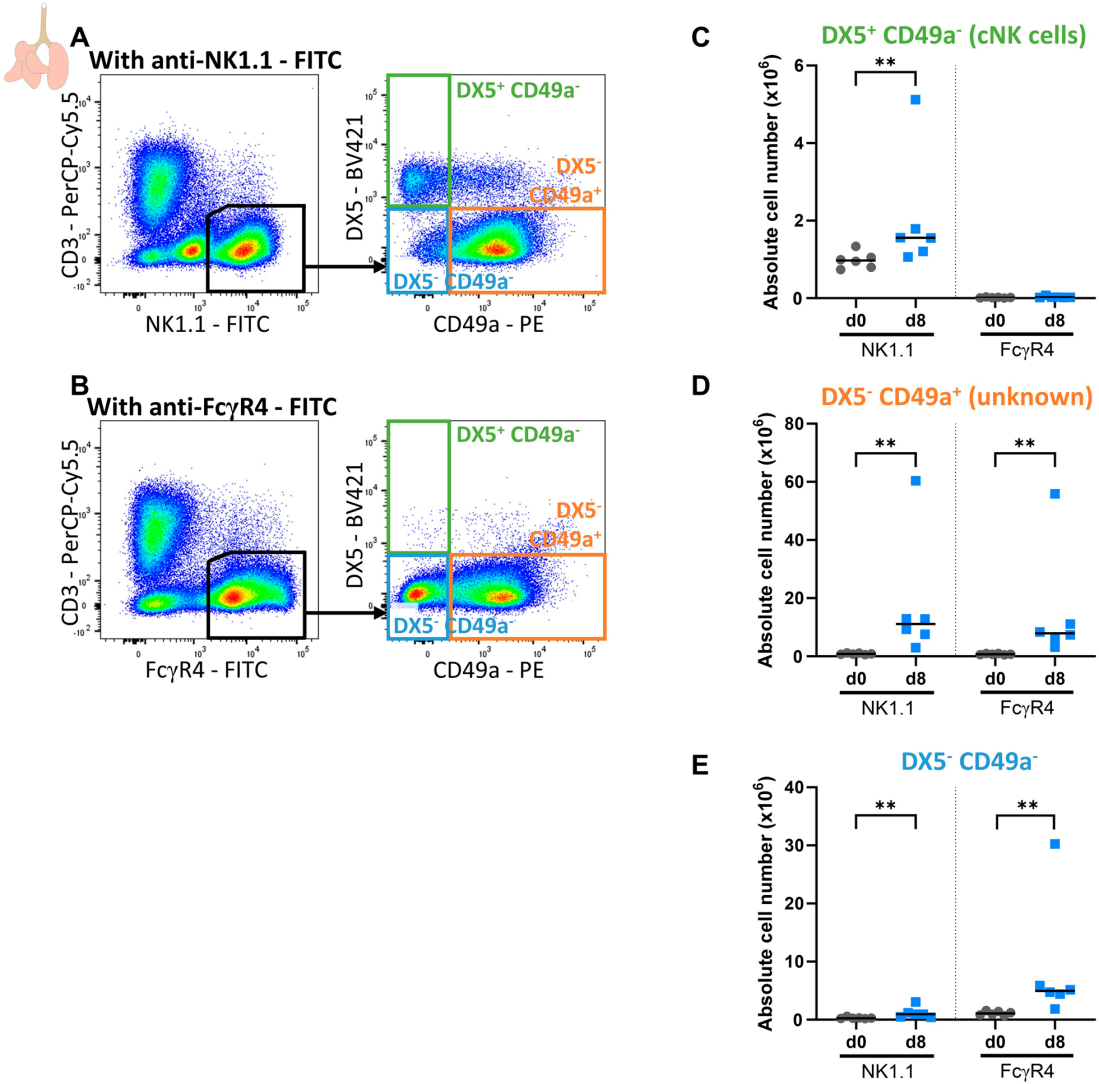
Aspecific binding of anti-NK1.1 antibodies may be mediated by Fc receptors, such as FcγR4. C57BL/6 mice were infected with *P. berghei* NK65 and dissected at 8. Leukocytes were isolated from the lungs according to protocol 2 and flow cytometry was performed. (**A**-**B**) Representative plots showing the gating of cells based on NK1.1 **A** or FcγR4 **B**. Absolute numbers of DX5^+^ CD49a^−^ (**C**), DX5^−^ CD49a^+^ (**D**) and DX5^−^ CD49a^−^ (**E**) cells on CD3^−^ NK1.1^+^ cells (left) or CD3^−^ FcγR4^+^ cells (right). **C**–**E** Data from two experiments. Inclusion or exclusion of the outlier in de d8 group does not affect the conclusion from these graphs. Significance was tested between d0 and d8 of CD3^−^ NK1.1^+^ cells and between d0 and d8 of the CD3^−^ FcγR4^+^ cells. Each symbol represents data of an individual mouse. n = 6 for d0 and d8. P-values were indicated as follows: *p < 0.05, **p < 0.01, ***p < 0.001. The horizontal black line in each group indicates the median

Biburger et al*.* showed that murine FcγR4 is responsible for aspecific staining with the mouse anti-NK1.1 antibody [23]. Therefore, the mouse anti-NK1.1 antibody was replaced with an Armenian hamster anti-FcγR4 antibody in the flow cytometry staining panels to evaluate whether FcγR4 could be responsible for the aspecific binding of anti-NK1.1 (Fig. 5). Staining appeared similar between the two panels except for the absence of DX5^+^ CD49a^−^ cNK cells in the CD3^−^ FcγR4^+^ population (Fig. 5A, B). These data indicate FcγR4^+^ cells do not include DX5^+^ CD49a^−^ cNK cells, but contain similar numbers of DX5^−^ CD49a^+^ and DX5^−^ CD49a^−^ cells (Fig. 5C). Interestingly, part of the DX5^+^CD49a^+^ cells in the NK1.1^+^ stained population were also absent in the FcγR4^+^-stained cells (Fig. 5A, B), suggesting that these might be cNK cells that have upregulated their CD49a expression. The remaining DX5^+^CD49a^+^ cells, present in both the NK1.1^+^- and FcγR4^+^-stained populations, appear more related to the FcγR4^+^ monocyte/macrophage population. Furthermore, gating the DX5^−^ CD49a^−^ and DX5^−^ CD49a^+^ population from the CD3^−^ FcγR4^+^ population, resulted in similar or higher numbers compared to gating from the CD3^−^ NK1.1^+^ population, suggesting that FcγR4 contributes to the aspecific binding of mouse anti-NK1.1 staining antibody on this macrophage-like cell population (Fig. 5D, E).

### Several myeloid cell types upregulate FcγR4 expression upon *P. berghei* NK65 infection

**Fig. 6:**
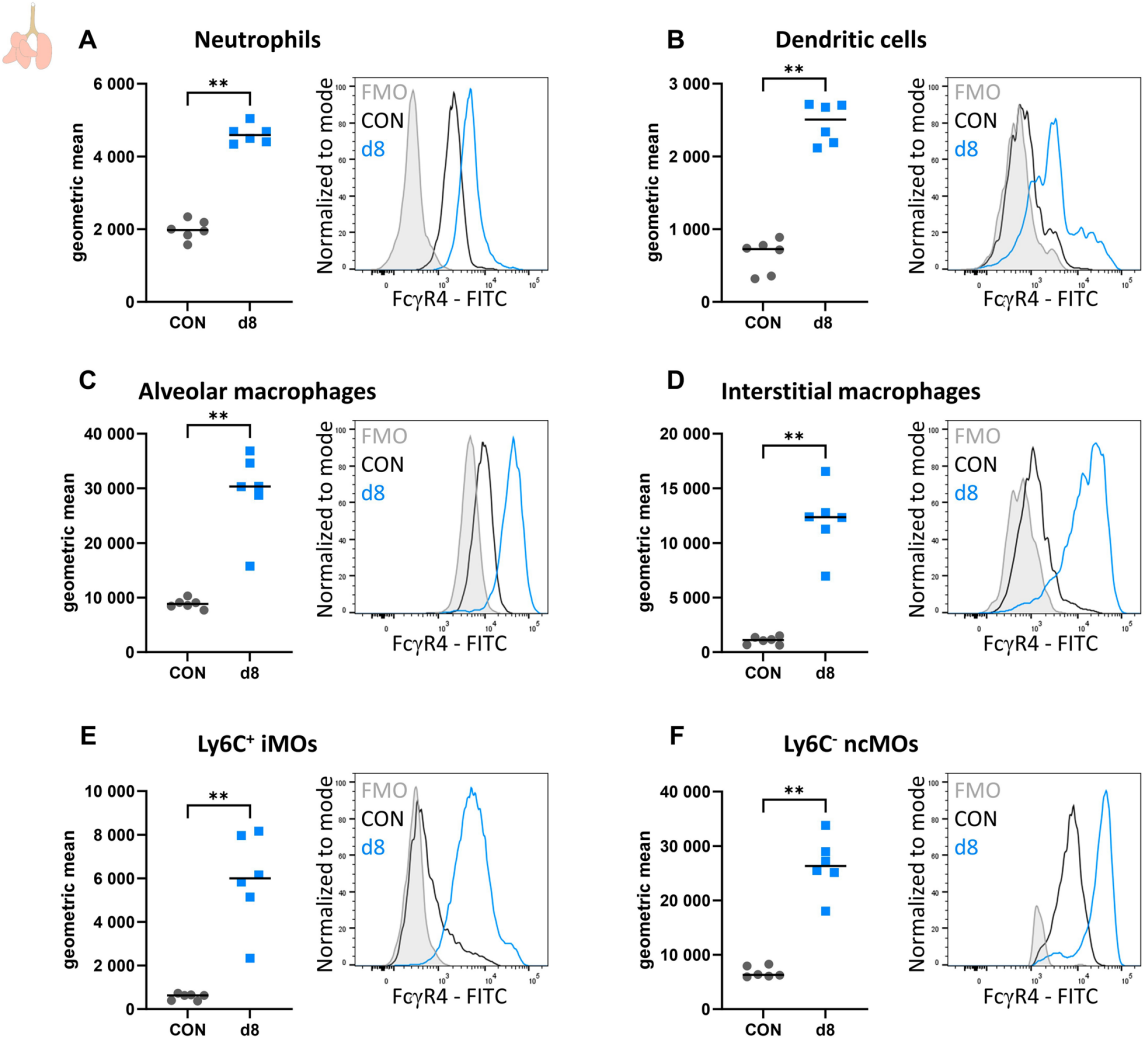
Several myeloid cell types upregulate FcγR4 expression upon *P. berghei* NK65 infection. C57BL/6 mice were infected with *P. berghei* NK65 and dissected at 8 dpi. Leukocytes were isolated from the lungs according to protocol 2 and flow cytometry was performed. The neutrophils (**A**; CD45^+^ Lin^−^ SiglecF^−^ CD11b^+^ Ly6G^+^), dendritic cells (**B**; CD45^+^ Lin^−^ SiglecF^−^ MHCII^+^ CD11c^+^), alveolar macrophages (**C**; CD45^+^ SiglecF^+^ CD11b^int^ CD11c^+^), interstitial macrophages (**D**; CD45^+^ Lin^−^ SiglecF^−^ Ly6G^−^ CD11b^hi^ MHCII^+^ CD24^−^ CD64^+^), Ly6C^+^ inflammatory monocytes (**E**; iMOs; CD45^+^ Lin^−^ SiglecF^−^ Ly6G^−^ CD11b^hi^ MHCII^−^ Ly6C^+^) and Ly6^−^ non-classical monocytes (**F**; ncMOs; CD45^+^ Lin^−^ SiglecF^−^ Ly6G^−^ CD11b^hi^ MHCII^−^ Ly6C^−^) in the lungs were gated and the intensity of FcγR4 was determined. Lineage gating was performed using CD3 and CD19. **A**–**F** Left graph: mean fluorescent intensity of FcγR4 was determined on each cell type. Each symbol represents data of an individual mouse. n = 6, for CON and d8. P-values were indicated as follows: *p < 0.05, **p < 0.01, ***p < 0.001. The horizontal black line in each group indicates the median. Right graph: representative histograms showing the normalized intensity of FcγR4 for the fluorescence minus one (FMO) control, an uninfected control mouse (CON) and a *P. berghei* NK65-infected mouse (d8)

To further investigate whether the FcγR4^+^ cells are myeloid cells, FcγR4 expression on various myeloid cell populations was analysed in lungs of uninfected control mice and *P. berghei* NK65-infected C57BL/6 mice at 8 dpi (Fig. 6). MFI and histogram plots for FcγR4 are shown for each myeloid cell population at the different time points with the corresponding FMO histogram plot as a negative control (Fig. 6). All myeloid cell populations upregulated FcγR4 expression upon infection at 8 dpi. Ly6C^−^ non-classical monocytes and alveolar macrophages were found to have the highest expression levels both in uninfected controls and in *P. berghei* NK65-infected mice at 8 dpi (Fig. 6C, F). However, as shown by the FMO, autofluorescence of the alveolar macrophages might influence the results (Fig. 6C).

These results indicate that several myeloid cells upregulate FcγR4 upon *P. berghei* NK65 infection at 8 dpi, which results in the aspecific binding of anti-NK1.1 antibodies.

## Discussion

In conclusion, cNK cells were dispensable in the development of MA-ARDS. Moreover, aspecific binding of mouse anti-NK1.1 staining antibodies to myeloid cells, such as monocytes and macrophages, was observed after infection with *P. berghei* NK65 via an upregulation of FcγR4 upon infection. These results demonstrate the importance of a critical mindset when analyzing flow cytometry results and the importance of checking for aspecific binding using isotype controls, even when using commercially available Fc blocking reagents. The use of anti-DX5 or anti-NKp46 antibodies is proposed as solution when analysing cNK cells using flow cytometry, since no aspecific binding of these antibodies was observed in the unknown population after malaria infection. This may be due to the fact that the anti-NK1.1 (clone PK136) is a mouse anti-mouse antibody, which may result in an increased risk of aspecific binding in mice in comparison to non-mouse antibodies. Importantly, the in vivo depletion using mouse anti-NK1.1 antibodies was specific for cNK cells.

The role of cNK cells in the development of malaria pathology remains uncertain [13]. In the literature, the role of cNK cells in malaria in in vitro and in vivo models has mainly been studied by using two different depleting antibodies, namely the mouse anti-asialo GM1 and the anti-NK1.1 antibodies. In the past, the anti-asialo GM1 antibody was used frequently but showed depletion of other leukocyte populations, such as T cells, basophils and monocytes [24–27]. In contrast, the anti-NK1.1 antibody (clone PK136) was found to be more specific for cNK cells, but not all mouse strains express NK1.1 (e.g. BALB/c and DBA/2 mice). Generally in the malaria immune response, a role for cNK cells was found in liver-stage sporozoite-induced immunity [16, 28–32]. Multiple murine studies, using both anti-asialo GM1 and anti-NK1.1 antibodies found no role for cNK cells in anti-parasitic immune responses against blood-stage parasites [15, 31, 33, 34]. In contrast, some murine studies using either anti-asialo GM1 antibodies or immunocompromised mice, found a crucial role for cNK cells in immunity against the blood-stage parasite [16, 30–32, 35]. While Hansen et al*.* found a crucial role for cNK cells in experimental cerebral malaria using anti-asialo GM1 antibodies, this was not confirmed upon depletion with anti-NK1.1 antibodies or by using the knock-out approach of Lymphocyte antigen 49E, which is an inhibitory receptor on cNK cells [14, 18, 36]. Also in liver pathology, no role for cNK cells was found [19]. These last studies are corroborated by the findings in this study showing that cNK cell depletion using the anti-NK1.1 antibody had no effect on the development of MA-ARDS [18, 19, 36].

In this study, the appearance of an unknown NK1.1^+^ population in lungs after infection with *P. berghei* NK65 was demonstrated. More detailed characterization first suggested a phenotype similar to ILC1-like cells, since these cells were DX5^−^ CD49a^+^ and CD4^−^ CD8^−^. However, the cells in this population were found to be larger and more granular than cNK cells and ILCs. Moreover, this population was found to be NKp46^−^. This is in contrast to the study of Klose et al*.*, where a population of liver ILC1s was observed as being NKp46^+^[37]. In both organs, this population decreased early after infection with *P. chabaudi* AS and returned later after the spontaneous clearance of the parasites.

The unknown population was found to contain monocytes and macrophages that bind the anti-NK1.1 antibodies aspecifically at 8 dpi. The appearance of this population was observed upon infection and *P. berghei* NK65 infection was accompanied by an increased expression of FcγR4 on multiple myeloid cell populations. Therefore, it was hypothesized that the aspecific binding occurs via this FcγR4 and may additionally occur via other Fc receptors, despite the use of the commercial murine Fc blocking reagents during flow cytometry staining. These results are in agreement with the study of Biburger et al*.* who showed that FcγR4 is not blocked by classical blocking reagents in mice, resulting in the aspecific binding of mouse anti-NK1.1 antibodies [23]. Therefore, they suggest the addition of FcγR4 blocking antibodies to common blocking strategies to obtain a wider inhibition of all types of Fcγ receptors. Since the aspecific binding was specific for mouse anti-NK1.1 antibodies and did not occur when using rat anti-DX5 antibodies or rat anti-NKp46 antibodies for flow cytometry staining, the use of these antibodies is proposed as an alternative to the anti-NK1.1 antibody when performing flow cytometry for cNK cells.

Importantly, in vivo depletion using the mouse anti-NK1.1 antibodies was found to be specific for cNK cells, since no significant difference in the number of cells in the unknown population was observed after injection of anti-NK1.1 antibodies. This may suggest an inability of antibodies to induce opsonization or complement activation after aspecific binding.

An increased expression of FcγR4 upon *P. berghei* NK65 infection was observed, which is in line with the appearance of this population after infection. This observation suggests that, while this aspecific binding may be limited in naïve C57BL/6 mice, misinterpretations may occur in infectious models, such as malaria. Similarly to the previously demonstrated importance of careful gating analysis for neutrophil depletion in malaria and auto-inflammatory mouse models [38], the potential risk of aspecific binding of murine staining antibodies was shown during flow cytometry and was caused by an upregulation of Fc receptors upon infection with *Plasmodium* parasites.

### Supplementary Information

**Additional file 1.** Additional figures and tables.

## Data Availability

Not applicable.

## Abbreviations

BALF: Broncho-alveolar lavage fluid
CCL5: CC chemokine ligand 5
cNK cells: Conventional natural killer cells
Dpi: Days post infection
FMO: Fluorescence minus one
FSC: Forward scatter
IFN-γ: Interferon-γ
ILCs: Innate lymphoid cells
iMOs: Inflammatory monocytes
i.p.: Intraperitoneal
MA-ARDS: Malaria-associated acute respiratory distress syndrome
ncMOs: Non-classical monocytes
PABA: 4-Amino-benzoic acid sodium
SSC: Side scatter
TNF: Tumour necrosis factor
