# Supplementary material for: Aspecific binding of anti-NK1.1 antibodies on myeloid cells in an experimental model for malaria-associated acute respiratory distress syndrome
Source: Malar J. 2024 Apr 18;23:110. doi: 10.1186/s12936-024-04944-9 (PMC11025177; doi:10.1186/s12936-024-04944-9)
Supplement: Supplementary file 1 — Additional file 1. Additional figures and tables. [file 12936_2024_4944_MOESM1_ESM.docx]

**Additional file 1: Table S1. Antibodies used for flow cytometry**

| **Antigen** | **Fluorophore** | **Clone** | **Isotype** | **Company** |
| --- | --- | --- | --- | --- |
| **Lymphoid panel (100 000 cells read)** | | | | |
| CD3 | FITC | 145-2C11 | Armenian hamster IgG1 | eBioscience |
| CD8 | PerCP-Cy5.5 | 43-6.7 | Rat IgG2a | eBioscience |
| CD49a | PE | Ha31/8 | Armenian hamster IgG2a | BD |
| NK1.1 | PE-Cy7 | PK136 | Mouse IgG2a | eBioscience |
| Isotype | PE-Cy7 | eBM2a | Mouse IgG2a | eBioscience |
| CD4 | APC-eFluor 780 | RM4-5 | Rat IgG2a | eBioscience |
| DX5 | APC | DX5 | Rat IgM | eBioscience |
| B220 | BV786 | RA3-6B2 | Rat IgG2a | BD |
| CD45 | BUV395 | 30-F11 | Rat IgG2b | BD |
| Live/dead | Zombie Aqua™ Fixable Viability Kit |  |  | Biolegend |
| **NK cell panel (100 000 cells read)** | | | | |
| NK1.1 | FITC | PK136 | Mouse IgG2a | eBioscience |
| CD3 | PerCP-Cy5.5 | 145-2C11 | Armenian hamster IgG1 | eBioscience |
| CD49a | PE | Ha31/8 | Armenian hamster IgG2a | BD |
| TRAIL | PE-Cy7 | N2B2 | Rat IgG2a | Biolegend |
| CXCR6 | APC | DANID2 | Rat IgG2a | Biolegend |
| DX5 | BV421 | DX5 | Rat IgM | BD |
| NKp46 | BV785 | 29A1.4 | Rat IgG2a | Biolegend |
| Live/dead | Zombie Aqua™ Fixable Viability Kit |  |  | Biolegend |
| **Competition test anti-NK1.1 & isotype control (100 000 cells read)** | | | | |
| NK1.1 | FITC | PK136 | Mouse IgG2a | eBioscience |
| CD49a | PE | Ha31/8 | Armenian hamster IgG2a | BD |
| Isotype | PE-Cy7 | eBM2a | Mouse IgG2a | eBioscience |
| DX5 | APC | DX5 | Rat IgM | eBioscience |
| CD3 | BV650 | 17A2 | Rat IgG2b | Biolegend |
| CD45 | BUV395 | 30-F11 | Rat IgG2b | BD |
| Live/dead | Zombie Aqua™ Fixable Viability Kit |  |  | Biolegend |
| **Panel for sorting (150 000 cells sorted per population)** | | | | |
| CD3 | FITC | 145-2C11 | Armenian hamster IgG1 | eBioscience |
| CD11b | PerCP-Cy5.5 | M1/70 | Rat IgG2b | eBioscience |
| CD49a | PE | Ha31/8 | Armenian hamster IgG2 | BD |
| NK1.1 | PE-Cy7 | PK136 | Mouse IgG2a | eBioscience |
| MHCII | APC | M5/114.15.2 | Rat IgG2b | Biolegend |
| DX5 | BV421 | DX5 | Rat IgM | BD |
| CD45 | BUV395 | 30-F11 | Rat IgG2b | BD |
| Live/dead | Fixable Viability dye eFluor™ 780 |  |  | eBioscience |
| **Lymphoid panel (with or without FcγR4) (200 000 cells read)** | | | | |
| NK1.1 | FITC | PK136 | Mouse IgG2a | eBioscience |
| FcγR4 | FITC | 9E9 | Armenian hamster IgG | Biolegend |
| CD49a | PE | Ha31/8 | Armenian hamster IgG2 | BD |
| CD45 | APC-Cy7 | 30-F11 | Rat IgG2b | Biolegend |
| DX5 | BV421 | DX5 | Rat IgM | BD |
| CD3 | BUV395 | 145-2C11 | Armenian hamster IgG1 | BD |
| Live/dead | Zombie Aqua™ Fixable Viability Kit |  |  | Biolegend |
| **Myeloid panel with FcγR4 (200 000 cells read)** | | | | |
| FcγR4 | FITC | 9E9 | Armenian hamster IgG | Biolegend |
| CD11c | PE-Cy7 | N418 | Armenian hamster IgG | Biolegend |
| CD64 | PE | X54-5/7.1 | Mouse IgG1 | Biolegend |
| CD24 | PE-CF594 | M1/69 | Rat IgG2b | Biolegend |
| SiglecF | eFluor 660 | 1RNM44N | Rat IgG2a | eBioscience |
| Ly6G | Alexa fluor 700 | 1A8 | Rat IgG2a | BD |
| Ly6C | APC-Cy7 | AL-21 | Rat IgM | BD |
| CD11b | eFluor 450 | M1/70 | Rat IgG2b | eBioscience |
| MHCII | Horizon v500 | M5/114.15.2 | Rat IgG2b | BD |
| CD3 | BV650 | 17A2 | Rat IgG2b | Biolegend |
| CD19 | BV650 | 6D5 | Rat IgG2a | Biolegend |
| Live/dead | Zombie UV™ Fixable Viability Kit |  |  | Biolegend |

**Additional file 1: Figure S1**


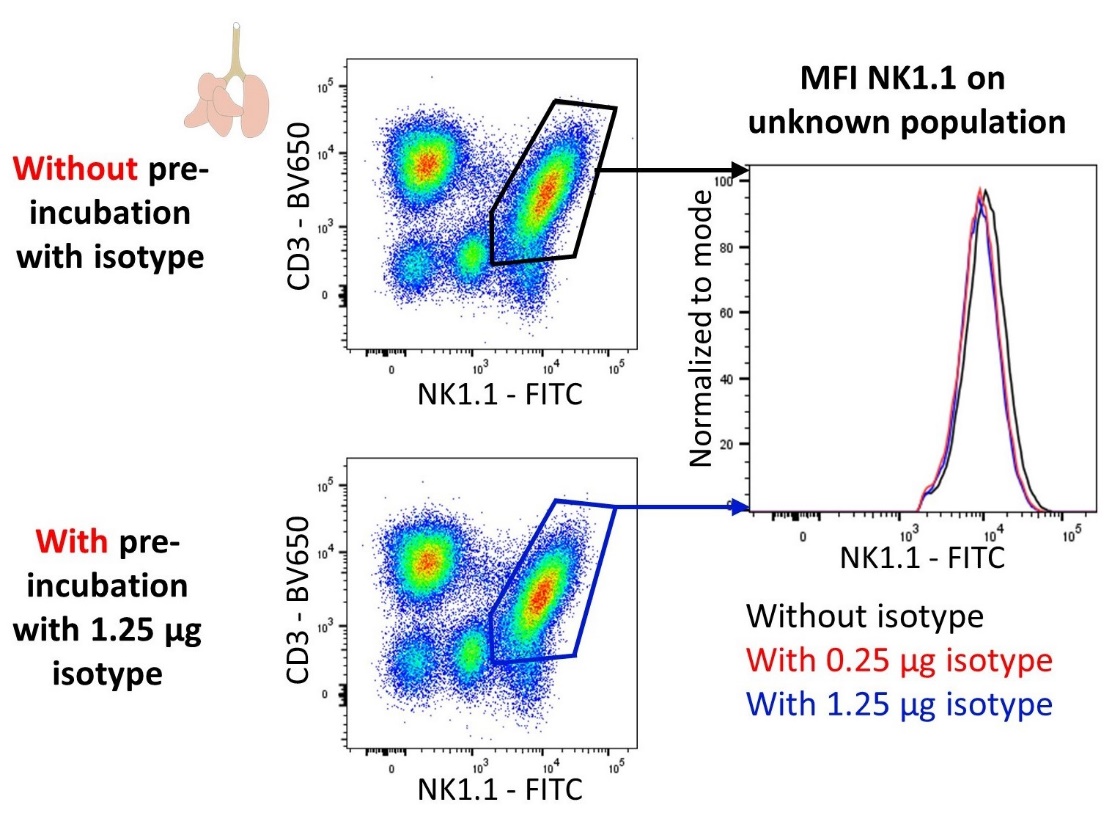


**Additional file 1: Figure S1. Competition test between the isotype control and anti-NK1.1 antibody showed no competition at the concentrations used.** C57BL/6 mice were infected with *Pb*NK65. Mice were dissected at 9 dpi. Leukocytes were isolated from the lungs according to protocol 2 and flow cytometry was performed. Cells were pre-incubated with 0.25 µg or 1.25 µg of isotype antibodies before staining with 0.25 µg of anti-NK1.1 antibodies to check for competition. Determination of the mean fluorescent intensity (MFI) of NK1.1 on the unknown population after pre-incubation with isotype antibodies or not.
